# Coumarins from *Angelica Lucida* L. - Antibacterial Activities

**DOI:** 10.3390/molecules14082729

**Published:** 2009-07-27

**Authors:** Jaroslaw Widelski, Milena Popova, Konstantia Graikou, Kazimierz Glowniak, Ioanna Chinou

**Affiliations:** 1 Department of Pharmacognosy with Medicinal Plant Laboratory, Skubiszewski Medicinal University of Lublin, 1 Chodzki str., 20-093, Poland; E-mails:yarpen222@interia.pl (J.W.), kglowniak@pharmacognosy.org (K.G.); 2 Institute of Organic Chemistry with Centre of Phytochemistry, Bulgarian Academy of Sciences, Acad. G. Bonchev str., bl.9, 1113 Sofia, Bulgaria; E-mail: popova@orgchm.bas.bg (M.P.); 3 Division of Pharmacognosy and Chemistry of Natural Products, Faculty of Pharmacy, University of Athens, Athens 15771, Greece; E-mail: kgraikou@pharm.uoa.gr (K.G.)

**Keywords:** *Angelica lucida*, apiaceae, coumarins, antibacterial activity

## Abstract

The first phytochemical investigation of the fruits of *Angelica lucida* has led to the isolation and characterization of five known coumarins (imperatorin, isoimperatorin, heraclenol, oxypeucedanin hydrate and heraclenin). All isolated compounds were identified by means of spectral and literature data. The extracts and the isolated constituents from *A. lucida* have been also evaluated for their antimicrobial activity against six Gram positive and negative bacteria, two oral pathogens and three human pathogenic fungi, exhibiting an interesting antimicrobial profile.

## Introduction

*Angelica lucida L.* is a species of *Angelica* genus (Apiaceae) known by the common names seacoast angelica and sea-watch. This plant is generally similar in appearance to other *Angelicas* with tall, dense umbels of yellowish-white flowers. As its common names suggest, this plant is found most often along the coastline. Its distribution includes the east and west coasts of North America, plus parts of East Asia. Tender parts of the plant have been used by the Eskimos as food, analgesic and tonic against common colds. The roots as well as the young stems of the plant have been taken as a preventative medicine [1]. The presence in the genus *Angelica* of coumarins [2], which are known for a broad spectrum of pharmacological properties [3], is well documented This evidence together with our research interests concerning the chemical composition of different Apiaceae (Umbeliferae) plants, prompted us to study the chemical composition of the title plant. Our study of *Angelica lucida* fruits has led to the isolation and characterization of five known coumarins. These compounds have been also isolated from other species of the genus *Angelica* [4,5,6], but for the first time to our knowledge from *A. lucida*, while terpenes have been previously referred from the essential oil of the seeds [7].

## Results and Discussion

**Figure 1:**
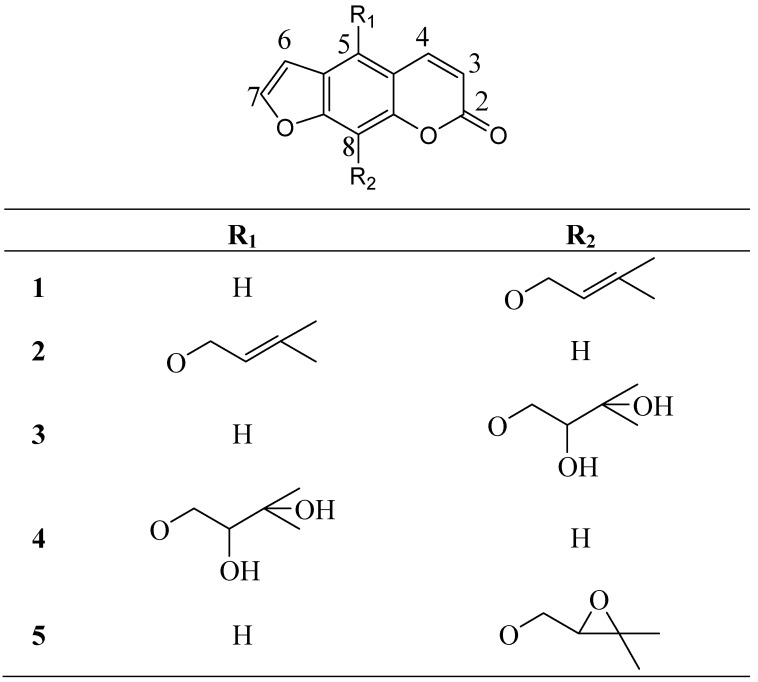
The structures of compounds **1**-**5.**

Examination of the petroleum ether and methanolic extracts of *A. lucida* fruits led to the isolation of five known coumarins: imperatorin (**1**) [4], isoimperatorin (**2)** [4], heraclenol (**3**) [4], oxypeucedanin hydrate (**4**) [4] and heraclenin (**5**) [8] (Figure 1). To the best of our knowledge, all determined coumarins, are reported for the first time from the studied plant. Constituents **1**-**4** have been also isolated from a quite big number of other *Angelica* species, and could be characterized as a chemotaxonomic tool of the genus, while heraclenin (**5**) has been previously isolated only from three Angelica taxa: *A. ursine* [9]*, A. genuflexa* [10] and *A. silvestris* [11]. The results of our study support the conclusion that coumarins are among the most characteristic and distinguishable chemical markers of the genus, as it has been previously mentioned, as well as of the Apiaceae family [1,2,12,13].

### Antimicrobial activity

The tested material was the petroleum ether and methanolic extracts as well as the isolated coumarins. The crude extracts of the plant were moderately active against Gram positive and negative bacteria and oral pathogens, while the tested extracts and isolated compounds were inactive against the three assayed *Candida* species. All isolated coumarins have been studied, to the best of our knowledge, for the first time against the oral pathogens *S. mutans* and *S. viridans* and generally they inhibited all assayed bacterial strains (MIC between 0.012 and 0.85 mg/mL) [12,13,14] while **1** and **2** appeared as the most active (MICs 0.012 and 0.70 mg/mL). This is in agreement with the observations of Schinkovitz *et al*. [12] who found that the presence of an isoprenyl unit attached to the carbocyclic ring favors their antimicrobial activities and Tada *et al*. [13], who showed a broad spectrum of activities of several similar furanocoumarins and their glucosides. Both Gram positive and negative bacteria displayed the same sensitivity to tested coumarins, while *P. aeruginosa* appeared as the most resistant strain, which as a result is in accordance with previous scientific results from Rosselli *et al.* [14].

The results of our antibacterial studies could be attributed to the coumarin ring as such natural products are known since the 1950s to exert their effects by inhibition of bacterial nucleic acid synthesis [14]. It has been proved that the addition of a prenyl group to the furanocoumarin skeleton results in an increase in lipophilicity of the molecule, facilitating its passage though the thick bacterial membrane to its target [15].

## Experimental

### General

NMR spectra were recorded in CDCl_3_ on a Bruker AC 200 at 50 MHz and on a Bruker Avance 400 at 400 MHz and the UV spectra on a Shimadzu UV-160A spectrophotometer.

### Plant material

*Angelica lucida* L. (Apiaceae) fruits were collected from the Botanical Garden of the Department of Pharmacognosy (Medical University of Lublin, Poland) in September of 2006 (voucher specimen n. 2534). Seeds for cultivation were provided by Dr Toshiro Shibata (Research Centre for Medicinal Plant Resources) from Coast line of Hamatonbetsutyo, Esashigun, Hokkaido, Japan (ca 142°22′E, 45°7′N) and were also identified botanically by him.

### Extraction and isolation

The air-dried aerial parts of the plant were pulverized and extracted with petroleum ether and methanol. Their petroleum ether and methanolic residues were subjected to chromatographic separations, mainly by column LC (VLC, CC). By these means, five coumarins were isolated: imperatorin (**1, **51.0 mg/41.3 mg isolated from the petroleum ether extract plus 9.7 mg from the methanolic one), isoimperatorin (**2, **31.7 mg), heraclenol (**3, **9.4 mg), oxypeucedanin hydrate (**4, **21.8 mg) and heraclenin (**5**, 17.7 mg). Their structures were established by spectroscopic methods including 1D- and 2D-NMR experiments.

**Table 1:** Antimicrobial activities MIC values (mg/mL, n=3) of the assayed extracts and isolated compounds.

	*S. aureus*	*S. epidermidis*	*P. aeruginosa*	*E. cloacae*	*K. Pneumoniae*	*E. coli*	*S. mutans*	*S. viridans*	*C. albicans*	*C. tropicalis*	*C. glabrata*
*petroleum ether extract*	3.40 ± 0.4	3.80 ±0.5	3.75 ± 0.4	3.64 ± 0.6	4.10 ± 0.7	2.97 ±0.3	2.68 ± 0.3	2.35 ±0.4	inactive	inactive	inactive
*methanolic extract*	1.97 ± 0.3	1.85 ± 0.2	2.28 ± 0.3	2.90 ±0.4	2.76 ±0.2	2.39 ±0.3	2.20 ±0.2	2.47 ±0.2	inactive	inactive	inactive
*imperatorin*	0.045 ±0.1	0.035 ±0.2	0.070 ±0.1	0.028 ±0.2	0.030 ±0.3	0.025 ±0.2	0.018 ±0.2	0.015 ±0.1	inactive	inactive	inactive
isoimperatorin	0.040 ±0.2	0.032 ±0.1	0.070 ±0.3	0.025 ±0.2	0.030 ±0.2	0.023 ±0.1	0.015 ±0.1	0.012 ±0.2	inactive	inactive	inactive
heraclenol	0.68 ±0.3	0.64 ±0.2	0.70 ±0.2	0.77 ±0.1	0.85 ±0.4	0.67 ±0.3	0.53 ±0.2	0.50 ±0.2	inactive	inactive	inactive
oxypeucedanin hydrate	0.65 ±0.3	0.60 ±0.2	0.81 ±0.3	0.80 ±0.4	0.72 ±0.3	0.65 ±0.2	0.57 ±0.2	0.55 ±0.2	inactive	inactive	inactive
heraclenin	0.80 ±0.2	0.82 ±0.1	0.85 ±0.4	0.85 ±0.6	0.77 ±0.2	0.70 ±0.5	0.75 ±0.2	0.83 ±0.4	inactive	inactive	inactive
netilmicin	0.004±0.1	0.004 ±0.1	0.088 ±0.3	0.008 ±0.2	0.008 ±0.2	0.010 ±0.1	nt	nt	nt	nt	nt
amoxicillin with clavulanic acid	0.003 ±0.2	0.003 ±0.1	0.003 ±0.2	0.042 ±0.2	0.048 ±0.3	0.005 ±0.1	nt	nt	nt	nt	nt
5-flucytocine	nt	nt	nt	nt	nt	nt	nt	nt	0.1x10^-3 ^±0.1	1x10^-3^ ±0.2	10x10^-3 ^±0.1
amphotericin B	nt	nt	nt	nt	nt	nt	nt	nt	1x10^-3 ^±0.3	0.5x10^-3 ^±0.1	0.4x10^-3 ^±0.1
sanguinarine	nt	nt	nt	nt	nt	nt	0.015 ±0.1	0.015 ±0.2	nt	nt	nt

nt: not tested.

### Antimicrobial activity assays

Antimicrobial activity against bacteria, oral pathogens and fungi was determined using the agar dilution technique [16]. For all assays, stock solutions of the tested extract and pure compounds in sterile distilled water with 10% Tween 80 have been prepared at 10 and 1 mg/mL, respectively. Serial dilutions of the stock solutions in broth medium (100 μL of Müller-Hinton broth, Sabouraud broth for fungi and blood agar 10% for oral pathogens) were prepared in a microtiter plate (96 wells). Then 1 μL of the microbial suspension (the inoculum, in sterile distilled water) was added to each well. For each strain, the growth conditions and the sterility of the medium were checked and the plates were incubated at 37 °C and the MICs were determined as the lowest concentrations preventing visible growth. 

### Microorganisms

A panel of microorganisms, including two Gram positive bacteria: *Staphylococcus aureus* (ATCC 25923) and *S. epidermidis* (ATCC 12228); four Gram negative bacteria: *Escherichia coli* (ATCC 25922), *Enterobacter cloacae* (ATCC 13047), *Klebsiella pneumoniae* (ATCC 13883) and *Pseudomonas aeruginosa* (ATCC 227853); three pathogenic fungi: *Candida albicans* (ATCC 10231), *C. tropicalis* (ATCC 13801) and *C. glabrata* (ATCC 28838) and the oral pathogens *Streptococcus mutans and S. viridans*, were used. Standard antibiotics (netilmicin and amoxicillin with clavulanic acid) were used in order to control the sensitivity of the tested bacteria and 5-flucytocine, amphotericin B and sanguinarine were used in order to control the tested fungi and the oral pathogens [16]. 

### Statistical analysis

Variance analysis of the results was taken using averages SD. Each value is the mean of three replications.
